# Crystallization and hardening of poly(ethylene-co-vinyl acetate) mouthguards during routine use

**DOI:** 10.1038/srep44672

**Published:** 2017-03-15

**Authors:** Ryoko Kuwahara, Ryotaro Tomita, Natsumi Ogawa, Kazunori Nakajima, Tomotaka Takeda, Hiroki Uehara, Takeshi Yamanobe

**Affiliations:** 1 Division of Molecular Science, Graduate School of Science and Technology, Gunma University, Kiryu, Gunma, Japan; 2 Department of Oral Health and Clinical Science, Division of Sports Dentistry, Tokyo Dental College, Chiyoda-ku, Tokyo, Japan

## Abstract

Mouthguards (MGs) made from poly(ethylene-co-vinyl acetate) (EVA) are widely used in contact sports to prevent injuries such as breaking teeth and lip lacerations and to reduce brain concussion. However, the changes in morphology and the molecular mobility of EVA, which can affect its physical properties during practical usage, have not been precisely examined. Therefore, we attempted to determine the main factors which lead to changes in MG performance after one season of practical use by high school rugby players. Solid-state nuclear magnetic resonance (NMR) and pulse NMR measurements showed the hardening of MGs, which was associated with an increased crystallinity of the EVA resulting from prolonged usage. Furthermore, our data indicated that the increase in the relative amount of the crystalline phase may be primarily attributed to temperature fluctuations and repeated changes in pressure, which could cause the hardening of EVA and eventually diminish the protective ability of MGs.

Mouthguards (MGs) can prevent sports-related oral injuries and reduce concussions, therefore, they are recently being employed for various sports^1^,^2^,^3^,^4^,^5^,^6^,^7^,^8^,^9^,^10^,^11^,^12^. Materials employed for MGs are very limited, and only poly(ethylene-co-vinyl acetate) (EVA), olefin-based thermoplastic elastomers and styrene-based thermoplastic elastomers have been certified in Japan^13^,^14^,^15^,^16^,^17^,^18^. EVA is primarily used because of its low cost and facile processability—the melting point of EVA (*ca*. 30–80 °C) is particularly low, for example^19^. Indeed, EVA sheets can easily be treated with commercially available small MG manufacturing machines; therefore, EVA MGs can be prepared not only at dental clinics but also at sporting grounds.

MGs are prepared by melting EVA sheets and subsequent molding with dental casts. MGs are detached from the molds after the temperature reaches room temperature. Following this, MGs undergo final occlusal checking by dentists before they are supplied to users. After MGs are used for long periods of time, some users report discomfort; in particular, they feel that their MGs are becoming hard. If hardening occurs, along with increased brittleness and reduced energy absorption capability, deterioration in the protective ability of the MG will occur concomitantly. To ensure that MGs are providing adequate safety levels to users, the guidelines for renewing MGs should preferably be based on scientific indicators.

Because EVA is a macromolecule with entangled polymer chains comprising crystalline and amorphous phases^20^,^21^, we presumed that the discomfort relating to the fit of MGs would mainly be derived not from chemical degradation but from the state of those phases, which may be influenced by temperature fluctuations and/or repeated changes in pressure. In this study, we precisely analysed routinely used MGs and EVA films using differential scanning calorimetry (DSC), solid-state NMR and pulse NMR measurements^22^,^23^,^24^,^25^,^26^,^27^ to identify the factors that affect MG morphology and molecular mobility.

## Results and Discussion

### NMR spectroscopic analyses of MGs after one season of use

**Figure 1:**
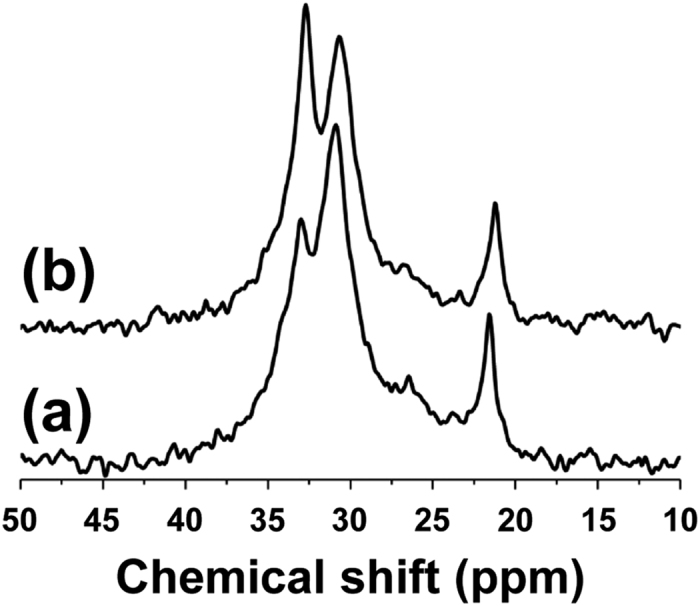
Solid-state CP/MAS ^13^C NMR spectra of (**a**) a piece from MGφ1 and (**b**) the piece of MG1 that contacts on tooth#16.

The solid-state cross-polarization magic-angle spinning (CP/MAS) ^13^C NMR spectra of a piece from MGφ1 and another from MG1 that contact on tooth#16, which is one of the most compressed occlusion parts of the MG, are displayed in Fig. 1. The notation MG1 represents a MG that was routinely used by user 1 for one season (10 months), whereas MGφ1 represents the excess portions of the MG material, which were obtained after the lamination and subsequent trimming of MG1 and preserved at room temperature for one season. Peaks at approximately 33 and 31 ppm are known to result from CH_2_ units in the ethylene groups of the crystalline and amorphous phases, respectively, whereas the peak corresponding to CH_3_ in the methyl group of acetate appears at 22 ppm^20^,^21^. As shown in Fig. 1a, the intensity of the peak corresponding to the amorphous phase was higher than that corresponding to the crystalline phase. Conversely, in Fig. 1b, the intensity of the peak corresponding to the crystalline phase was higher than that corresponding to the amorphous phase. Such phenomena were observed in all the MGs examined in this study (Table 1). These results indicate that the usage of MGs can increase the ratio of the crystalline phase present in EVA. Incidentally, the solution ^13^C NMR spectra of used MGs remained unchanged compared with those of unused MGs (data not shown), indicating that no chemical decomposition of EVA (e.g., hydrolysis of the acetate group)^28^ occurred during the usage period.

In solid-state CP/MAS ^13^C NMR, the efficiency of the cross-polarization from ^1^H to ^13^C in the crystalline phase is known to be higher than that in the amorphous phase. Therefore, in Fig. 1a, the ratio of the amount of the crystalline phase to that of the amorphous phase is, in fact, not as large as that indicated by the ratio of the two corresponding peaks. However, the change in the ratio of the peaks between unused and used MGs can be compared, and accordingly, the difference between the spectra (Fig. 1a,b) could be attributed to the crystallization during routine use.

We then assumed that the increase in the crystalline ratio may increase the fraction of restricted components in MGs. Pulse NMR measurements were therefore performed using the above-mentioned pieces of MGφ1–8 and MG1–8 to evaluate molecular mobility^29^,^30^. The observed data were fit to a hybrid of exponential and Gaussian functions to obtain the fraction ratios for the rigid, intermediate, and mobile components (Figure S1). As shown in Table 2, which summarizes the fractions of the rigid component for MGφ1–8 and MG1–8, the magnetization fractions demonstrate that MG usage increases the rigid component instead of decreasing the intermediate and mobile components, indicating a reduction in molecular mobility.

In summary, the CP/MAS ^13^C NMR and pulse NMR measurements show that the usage of EVA MG increases the crystalline fraction, which restricts molecular mobility and leads to the eventual hardening of the MG.

### Verification of the effect of temperature fluctuations by DSC

We attempted to verify the effects of temperature fluctuations on the crystallization behaviour of EVA films (0.030 cm thickness) with different vinyl acetate (VA) contents of 9%, 14% and 28%, for EVA9, EVA14 and EVA28, respectively, using DSC analysis. According to the solution ^13^C NMR spectra, the VA content of EVA28 was nearly equal to that of the EVA sheet (Drufosoft^®^) used to prepare the examined MGs.

**Figure 2:**
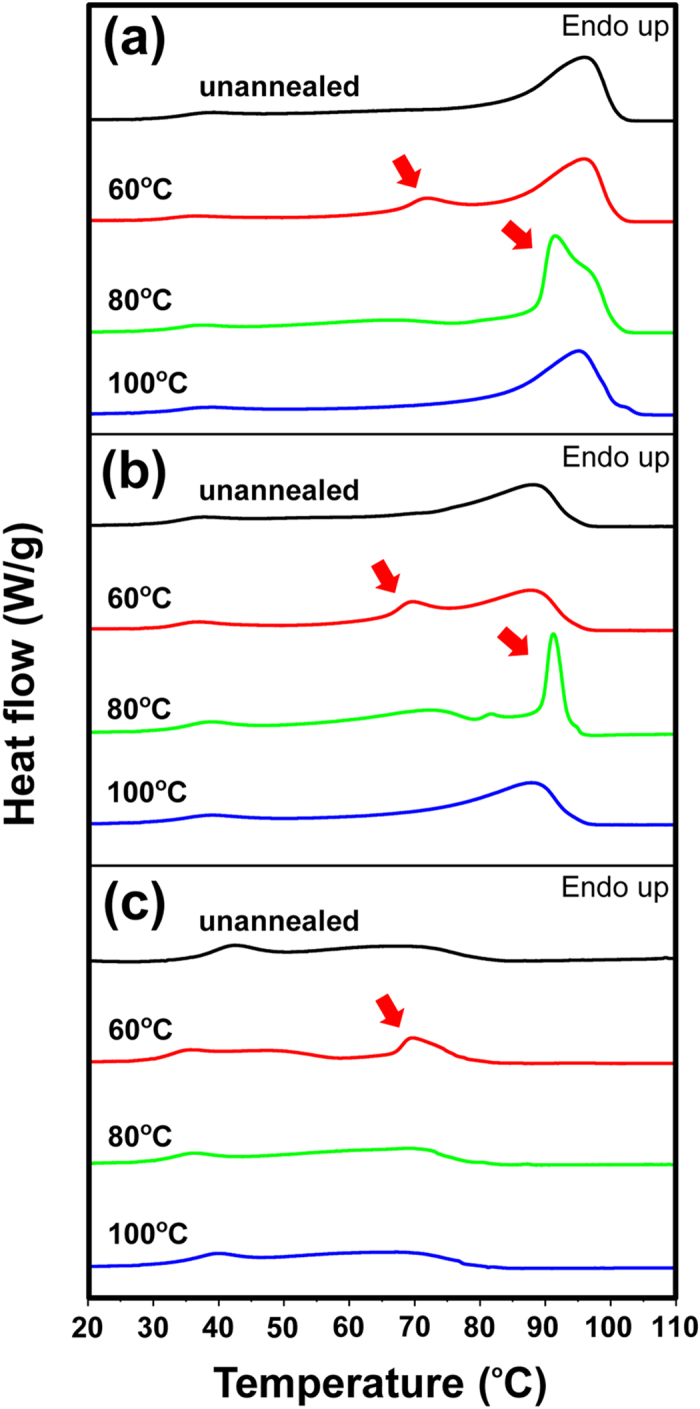
DSC curves of the heating process of (**a**) EVA9, (**b**) EVA14, and (**c**) EVA28. Samples: unannealed (black), annealed at 60 °C (red), annealed at 80 °C (green), and annealed at 100 °C (blue). New endothermic peaks were shown with red arrows.

Melting of unannealed EVA9 started at approximately 30 °C and ended after the stark endotherm at approximately 95 °C, as shown in the DSC curves of the heating process (Fig. 2a). In the cases of EVA14 and EVA28, melting started at approximately 30 °C; the former ended with a strong endotherm at approximately 90 °C, while the latter ended with a gently sloping approximately peak at approximately 70 °C (Fig. 2b,c). The decrease in the maximum intensity and temperature of the endothermic peaks depended on VA content, reflecting the thickness of lamellae^31^,^32^ mainly comprising ethylene units. Higher ethylene content, i.e. lower VA content, tended to provide thicker lamellae and enhance the crystallinity of EVA, although the broad melting ranges indicated that the lengths of the polyethylene strands between the VA units were widely distributed. Meanwhile, it can be stated that thin lamellae are produced regardless of the VA content because all films started melting at a low temperature (approximately 30 °C). This suggested that the crystallization of MG could progress in the mouth, i.e. at body temperature, during routine use.

Annealing of EVA9, EVA14, and EVA 28 resulted in a new gentle endothermic peak at approximately 70 °C when the films were annealed at 60 °C (Fig. 2a–c), and annealing of EVA9 and EVA14 at 80 °C resulted in a sharp endothermic peak at approximately 90 °C (Fig. 2a,b). Although the new endothermic peaks are attributed to the melting of the crystalline phase by the annealing treatments, it was commonly observed that annealing of the EVA films at the crystallization temperature (T_c_)^19^ or the closest temperature above T_c_ resulted in a relatively clear peak maximum for the melting temperature (T_m_), i.e. T_c_ = 80, 71 and 48 °C for EVA9, EVA14 and EVA28, respectively (Figs 2 and S2). Thus, these results indicate that the crystallinity of EVA MGs greatly depends on the status of the ethylene moieties and is significantly influenced by temperature fluctuations at ranges closer to T_c_.

**Figure 3:**
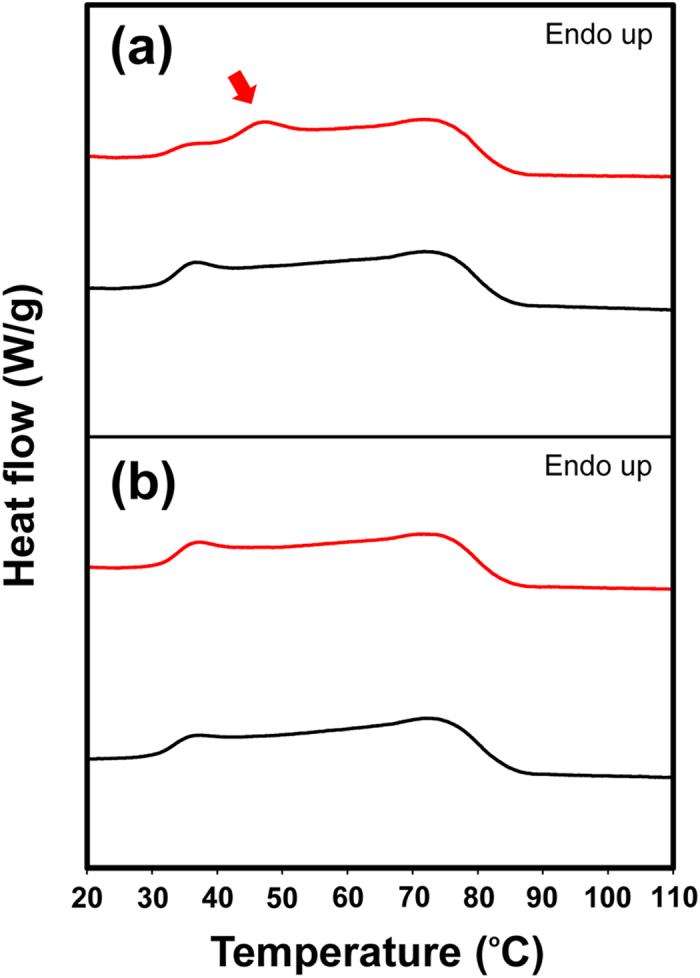
DSC curves of the heating process of Drufosoft^®^ films with or without treatment by thermal cycles (100 times) executed using a protocol with repeated temperature fluctuations (**a**) between 25 °C and 37 °C or (**b**) between 6 °C and 22 °C. Samples: thermal-treated (red) and as-prepared (black). The new endothermic peak is shown with a red arrow.

Furthermore, we analysed the effect of temperature fluctuations using Drufosoft^®^ films (thickness = 0.30 cm, which is approximately equal to that of typical MGs) with or without treatment of repeated thermal cycles (100 times). Two different protocols of thermal cycling were employed; one was shuttling between 25 °C and 37 °C and the other was shuttling between 6 °C and 22 °C as a reference condition (Fig. 3). Intriguingly, in the former condition, a new endothermic peak appeared at approximately 45 °C (Fig. 3a), but, in contrast, the latter condition caused little changes in the DSC thermogram (Fig. 3b). These results show that repeated temperature fluctuations even between close temperatures, i.e., ambient and body temperatures, can affect the crystallinity of EVA MGs.

### Verification of the effect of temperature fluctuations using solid-state CP/MAS ^13^C NMR

**Figure 4:**
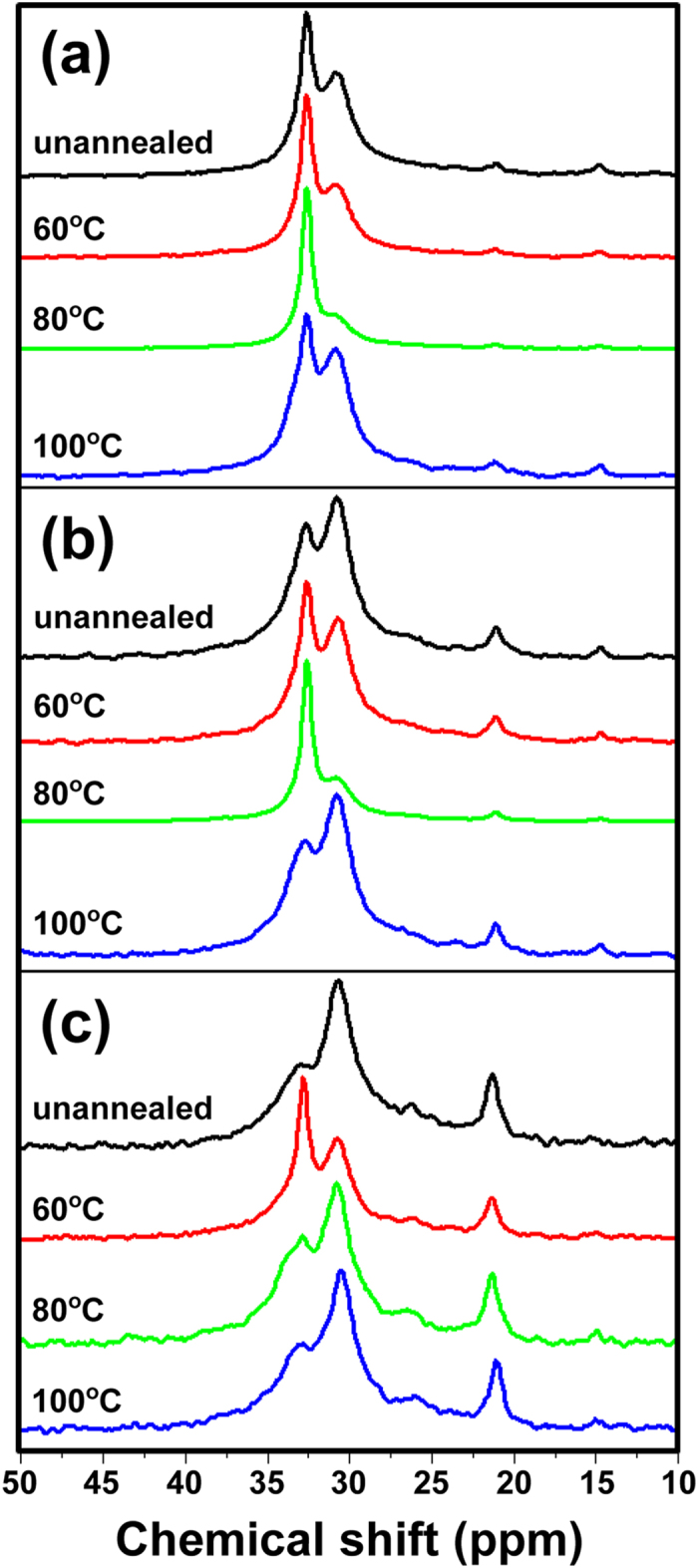
Solid-state CP/MAS ^13^C NMR spectra of (**a**) EVA9, (**b**) EVA14, and (**c**) EVA28. Samples: unannealed (black), annealed at 60 °C (red), annealed at 80 °C (green), and annealed at 100 °C (blue).

The tendency towards crystallinity discovered in the DSC measurements was also confirmed by solid-state CP/MAS ^13^C NMR analyses. As shown in Fig. 4a, although the intensity of the peak corresponding to the crystalline phase (at 33 ppm) was higher than that corresponding to the amorphous phase (at 31 ppm), the highest difference was observed in the spectrum of EVA9 annealed at 80 °C, which is equal to T_c_. Similarly, the highest differences were observed in EVA14 annealed at 80 °C (T_c_ = 71 °C) and in EVA28 annealed at 60 °C (T_c_ = 48 °C), respectively (Fig. 4b,c). Furthermore, intriguingly, when the spectra of unannealed EVA films were compared, the ease of crystallization obviously reflected the ethylene content.

### Verification of the effect of temperature fluctuations using pulse NMR

The molecular mobility in EVA films (0.030 cm thickness) was analysed using pulse NMR measurements. As compared with the unannealed EVA9 film, EVA9 films annealed at either 60 °C or 80 °C exhibited increased fraction ratios and decreased spin-spin relaxation times (T_2_ values) for the rigid component (Table S1). EVA9 annealed at 100 °C provided similar values to unannealed EVA9. Among the fraction ratios and T_2_ values obtained for the rigid component in all EVA9 films examined, EVA9 annealed at 80 °C showed maximum and minimum values (36.9% and 8.90 μs), respectively. In the case of EVA14, the maximum fraction ratio (30.6%) and the minimum T_2_ value (9.90 μs) of the rigid component were observed in films annealed at 80 °C. Furthermore, the rigid component of EVA28 annealed at 60 °C provided the minimum T_2_ value (11.0 μs), although the fraction ratio was equal to or somewhat smaller than the other EVA28 films. These results, showing the effects of temperature fluctuation on the hardening of EVA films, indicate that their hardening is well correlated with the crystallinity observed using DSC and solid-state CP/MAS ^13^C NMR measurements.

### Effects of repeated pressure on crystallization and compressive deformation behaviour

**Figure 5:**
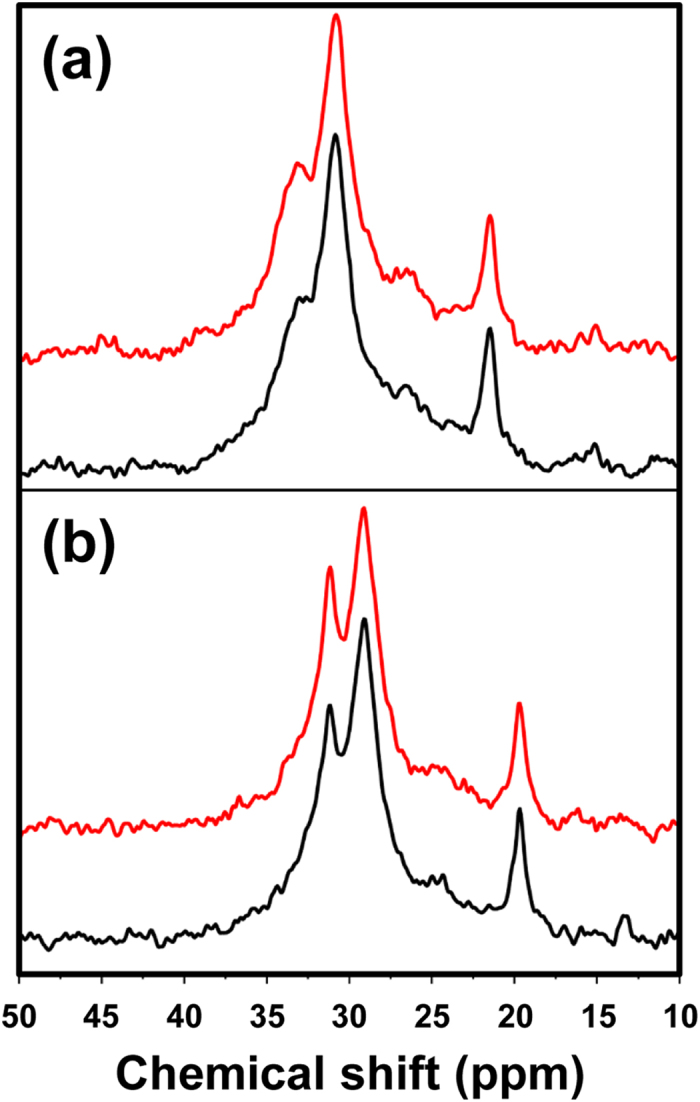
Solid-state CP/MAS ^13^C NMR spectra of (**a**) EVA28_1 and (**b**) EVA28_2 before (black) and after (red) repeated compression cycles (10,000 times at 6.0 MPa).

To study the changes in crystallinity resulting from repeated changes in pressure, EVA28 films (EVA28_1 and EVA28_2) were analysed by solid-state CP/MAS ^13^C NMR before and after repeated compression cycles (10,000 times at 6.0 MPa). As shown in Fig. 5a, the crystalline component of EVA28_1, corresponding to the signal at 33 ppm, seemed to increase slightly after compression (the ratios of the crystalline component in EVA28_1 before and after repeated compression cycles were 29.9% and 30.5%, respectively). This film was immediately subjected to cooling in ice cold water during its preparation. In contrast, an increase in the crystalline component after compression was clearly observed in EVA28_2, which was gradually cooled down to 25 °C during its preparation (the ratios of the crystalline component in EVA28_2 before and after repeated compression cycles were 32.0% and 35.5%, respectively) (Fig. 5b). Although both results indicate that repeated mechanical compression can induce the crystallization of MGs, the difference may be because EVA-28_2 originally contained a higher ratio of the crystalline component than EVA-28_1, as shown in the NMR spectra of the uncompressed films. In particular, the difference indicates that, in addition to repeated mechanical compressions, the presence of seed crystals could further accelerate crystallization within EVA MGs.

**Figure 6:**
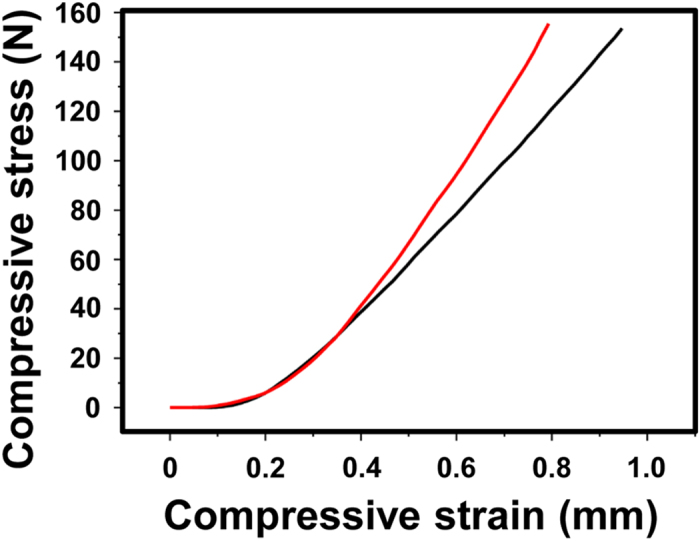
Compressive stress–strain curves of Drufosoft^®^ films (thickness = 0.30 cm) before (black) and after (red) repeated compression cycles (5,000 times at 6.0 MPa).

Finally, we analysed compressive deformation behaviour using Drufosoft^®^ films (thickness = 0.30 cm) before and after repeated compression cycles (5,000 times at 6.0 MPa). As shown in Fig. 6, compressive stress–strain curves clearly showed that the repeated compression resulted in a decrease in compressive strain *versus* compressive stress, indicating the decreased capacity of MG to withstand mechanical compressions.

## Conclusions

We focused on temperature fluctuations and repeated pressure changes as possible causes of deterioration in EVA MGs. To this end, we precisely analysed used MGs, Drufosoft^®^ and EVA films with different VA contents. The solid-state CP/MAS ^13^C NMR measurements showed significant increases in the crystalline components present in eight MGs after 10 months of routine use. These changes could generally be reproduced by heat annealing at temperatures around T_c_. Furthermore, pulse NMR measurements indicated that increased crystallinity results to the hardening of EVA materials, which may lead to a loss of the MGs’ protective ability. Generally, MGs are used at around body temperature (37 °C), which is lower than the T_c_ of EVA28 (48 °C); however, repeated cycles of temperature fluctuations between ambient and body temperatures could result in increased crystallinity and hardening because melting of this material starts at around 30 °C. In addition, EVA MGs should not be stored around or above T_c_, even for a short period of time.

Repeated mechanical compression (6.0 MPa, 10,000 times) also increased the crystallinity of EVA28 films, although the effect was not as large as the effect observed in the solid-state ^13^C NMR spectra of annealed samples. Finally, we verified the effect of repeated pressure changes using used EVA material, i.e. Drufosoft^®^ (6.0 MPa, 5,000 times) and observed decreased compressive strain *versus* compressive stress in the compressed film.

Our experimental data indicate that both temperature fluctuations and repeated pressure changes may affect the protective ability of EVA MGs, and we therefore suggest that changes in the crystallinity of EVA during routine use become one of the key scientific indicators of MG deterioration. Ideally, these changes should be measured by non-destructive methods, for e.g., using magnetic resonance imaging (MRI) apparatus, while the routinely used MGs were cut into pieces and analysed. However, MRI cannot be applied till now owing to technical limitations in resolution and signal/noise ratios^33^. Further development of NMR apparatus and/or application of other spectroscopic methodologies, such as terahertz (THz) imaging^34^,^35^, will enable non-destructive detection of the crystallinity of EVA MGs.

## Experimental Procedure

### Materials

Clear-transparent Drufosoft^®^ Type SQ EVA with a 3 mm thickness (Dreve-Dentamid GmbH, Unna, Germany) was used as the MG material. EVA pellets with various vinyl acetate (VA) contents, viz., 9, 14 and 28% VA, were purchased from Scientific Polymer Products, Inc. (NY, USA). In this study, films prepared from those pellets were named EVA9, EVA14 and EVA28, respectively.

### Preparations of MGs and EVA films

Using a dental pressure laminate machine, Drufomat SQ (Dreve-Dentamid GmbH, Unna, Germany), eight MGs were prepared from respective dental casts, which were formed from eight high school rugby players (also referred to as ‘users’). The excess portions of the respective MG materials (MGφx), which were obtained after the lamination and subsequent trimming of MGs, were preserved at room temperature as controls, whereas the prepared MGs (MGx) were used for one season (10 months), where x = user number (Tables 1 and 2). Methods were conducted according to the relevant guidelines and regulations. Informed consent was obtained from all the participants. The implementation plan of this study was approved by the Ethics Committee of Tokyo Dental College (Ethical Clearance No. 437).

EVA films, i.e. EVA9, EVA14 and EVA28, were prepared from the EVA pellets using a compression-molding machine, Table-type-test press SA-303-I-S (Tester Sangyo Co Ltd., Saitama, Japan). The pellets underwent compression molding at 230 °C and 45 MPa for 10 min followed by quick quenching in ice-cold water to yield the unannealed EVA films with a thickness of 0.30 or 0.030 cm. Annealed EVA films were prepared by reheating the unannealed films at 60, 80 or 100 °C for 60 min and subsequent quick quenching in ice-cold water. To confirm reproducibility, preparation and measurement of EVA films were performed in three independent experiments.

### ^13^C NMR measurements

The solution ^13^C NMR and the solid-state CP/MAS ^13^C NMR spectra were recorded on a Bruker AVANCE III spectrometer (Bruker BioSpin K.K., Kanagawa, Japan). The solution ^13^C NMR spectra were recorded at 75 MHz after samples were dissolved by soaking in chloroform overnight. Once the samples are dissolved, the crystalline phase is no longer retained. Therefore, the spectra can only reflect the changes in chemical structure but not those in polymer morphologies. For the solid-state CP/MAS ^13^C NMR, all samples were packed in a zirconia rotor with a 4 mm diameter and spun at 4 kHz in the instrument. The contact and repetition times were set at 2 ms and 5 s, respectively.

### Pulse NMR measurements

Pulse NMR measurements were made on a JEOL MU-25 spectrometer (JEOL Ltd., Tokyo, Japan). A solid-echo pulse sequence provided free induction decay curves, which were fitted using a hybrid of exponential and Gaussian functions (Figure S1)^29^, and in this way fraction ratios and T_2_ values could be determined (Tables 2 and S1).

### DSC measurements

DSC measurements were conducted on a Diamond DSC instrument (PerkinElmer Japan Co., Ltd., Kanagawa, Japan) calibrated with indium and tin standards^36^,^37^. The DSC scans were performed under a nitrogen atmosphere over a temperature range from 0 to 150 °C at a heating rate of 10 °C/min.

### Repeated thermal cycle experiments

Drufosoft^®^ films (thickness = 0.30 cm) were obtained by cutting the top face of box-type MG imitations (Figure S4), which were prepared as for the MGs using Drufomat SQ with a rectangular plaster cast instead of a cast constructed from a user. DSC curves were measured for the Drufosoft^®^ films with or without treatment by repeated thermal cycles (100 times) using a TC-312 thermal cycler (Techne Ltd., Cambridge, UK)^38^,^39^,^40^ (Fig. 3). Some samples were set into the thermal cycler (Figure S5) followed by thermal treatment with two different protocols (repeated temperature fluctuations between 25 °C for 60 min and 37 °C for 60 min, and between 6 °C for 60 min and 22 °C for 60 min) (Figure S3) prior to DSC measurements; the others were analysed by DSC without the thermal treatment.

### Repeated compression experiments

Two types of unannealed EVA28 films (length, width and thickness = 1.2 cm, 1.2 cm and 0.30 cm, respectively) underwent 10,000 repeated cycles of compression and release with 864 N of force (i.e. 6.0 MPa) using a Strograph E3-L (Toyo Seiki Seisaku-sho, Ltd., Tokyo, Japan) at room temperature. The first type of EVA film was prepared in compliance with the above-mentioned procedure to yield the EVA28_1 film. The second film type was prepared as follows: EVA28 pellets underwent compression molding at 230 °C and 45 MPa for 10 min followed by gradual overnight cooling to 25 °C in an incubator, M-230FN (Taitec Corporation, Saitama, Japan), to yield the EVA28_2 film. All samples were analysed by solid-state CP/MAS ^13^C NMR measurements.

### Compressive stress–strain measurements

Using a Strograph E3-L, compressive stress–strain curves were measured for Drufosoft^®^ films (length, width and thickness = 0.5 cm, 0.5 cm and 0.30 cm, respectively) before and after repeated cycles (5,000 times) of compression and release at 150 N (i.e. 6.0 MPa) and at room temperature. Drufosoft^®^ films were prepared as for the MGs using Drufomat SQ but using a rectangular plaster cast instead of a cast formed from a user.

## Additional Information

**How to cite this article:** Kuwahara, R. *et al*. Crystallization and hardening of poly(ethylene-co-vinyl acetate) mouthguards during routine use. *Sci. Rep.* **7**, 44672; doi: 10.1038/srep44672 (2017).

**Publisher's note:** Springer Nature remains neutral with regard to jurisdictional claims in published maps and institutional affiliations.

## Supplementary Material

Supplementary Information

## Figures and Tables

**Table 1:** Crystalline ratio (%) determined by solid-state CP/MAS ^13^C NMR spectroscopy.

MG1	MG2	MG3	MG4	MG5	MG6	MG7	MG8
44.1	42.5	37.9	39.7	41.6	42.1	36.9	41.2
**MGφ1**	**MGφ2**	**MGφ3**	**MGφ4**	**MGφ5**	**MGφ6**	**MGφ7**	**MGφ8**
33.0	35.0	34.6	32.7	34.4	38.0	33.5	35.9

**Table 2:** Rigid component (%) of MG determined using pulse NMR spectroscopy.

MG1	MG2	MG3	MG4	MG5	MG6	MG7	MG8
30.6	27.0	26.5	25.5	24.5	26.8	24.9	24.5
**MGφ1**	**MGφ2**	**MGφ3**	**MGφ4**	**MGφ5**	**MGφ6**	**MGφ7**	**MGφ8**
22.7	22.1	23.7	24.0	23.1	22.4	22.7	23.5
