# Supplementary material for: Crystallization and hardening of poly(ethylene-co-vinyl acetate) mouthguards during routine use
Source: Sci Rep. 2017 Mar 15;7:44672. doi: 10.1038/srep44672 (PMC5353674; doi:10.1038/srep44672)
Supplement: Supplementary Information [file srep44672-s1.pdf]

Supplementary Information

Crystallization and hardening of poly(ethylene-co-vinyl acetate) mouthguards during routine use

Ryoko Kuwahara[1], Ryotaro Tomita[1], Natsumi Ogawa[1], Kazunori Nakajima[2], Tomotaka Takeda[2], Hiroki Uehara[1], and Takeshi Yamanobe[1]

[1] Division of Molecular Science, Graduate School of Science and Technology, Gunma University, Kiryu, Gunma, Japan

[2] Department of Oral Health and Clinical Science, Division of Sports Dentistry, Tokyo Dental College, Chiyoda-ku, Tokyo, Japan

Correspondence: Professor T. Yamanobe, Gunma University

E-mail: yamanobe@gunma-u.ac.jp

Table S1 Effect of temperature fluctuations verified using pulse NMR spectroscopy.

Sample	Treatment	Unannealed		Annealed at 60°C		Annealed at 80°C		Annealed at 100°C	
	Fraction, T_2	Fraction (%)	T_2 (μs)	Fraction (%)	T_2 (μs)	Fraction (%)	T_2 (μs)	Fraction (%)	T_2 (μs)
EVA9	Rigid	34.9	9.80	36.1	9.20	36.9	8.90	34.3	9.80
	Intermediate	53.2	40.8	51.6	42.9	51.6	39.5	54.5	39.2
	Mobile	11.8	216	12.3	242	11.5	221	11.2	214
EVA14	Rigid	29.5	11.0	30.4	10.0	30.6	9.90	28.8	12.0
	Intermediate	56.4	45.4	54	47.8	55.8	43.4	59.0	41.9
	Mobile	14.1	242	15.6	262	13.6	233	12.2	216
EVA28	Rigid	20.3	12.5	19.3	11.0	21.1	12.4	21.5	12.5
	Intermediate	52.6	70.1	52.3	69.5	53.9	62.7	53.4	64.3
	Mobile	27.1	379	28.5	375	25.0	329	25.2	335

(a)

$$\boldsymbol{M(t) = A_r e^{-\frac{1}{2}\left(\frac{t}{T_{2r}}\right)^2} + A_i e^{-\frac{t}{T_{2i}}} + A_m e^{-\frac{t}{T_{2m}}}}$$

A_r: Fraction of the rigid component

A_i: Fraction of the intermediate component

A_m: Fraction of the mobile component

T_{2r}: T_2 value for the rigid component

T_{2i}: T_2 value for the intermediate component

T_{2m}: T_2 value for the mobile component

(b)

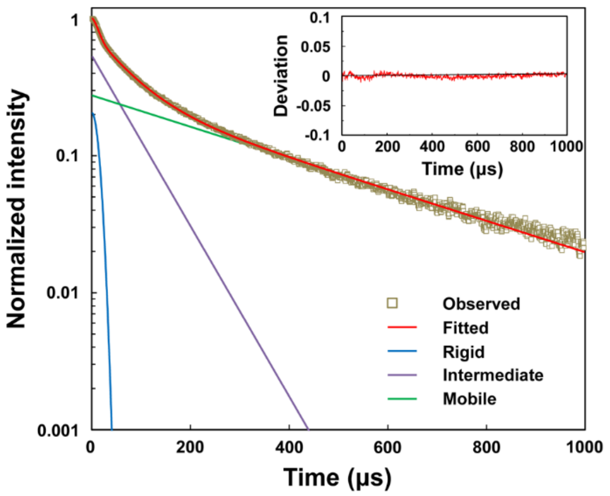

Figure S1. (a) The equation for the curve of best fit and (b) example of curve-fitting for a relaxation decay curve recorded with a drawing time of 1000 μs for the EVA sample, assuming three-component resolutions using the above-mentioned equation. The inset figure depicts the deviation of the fitting value from the experiment value.

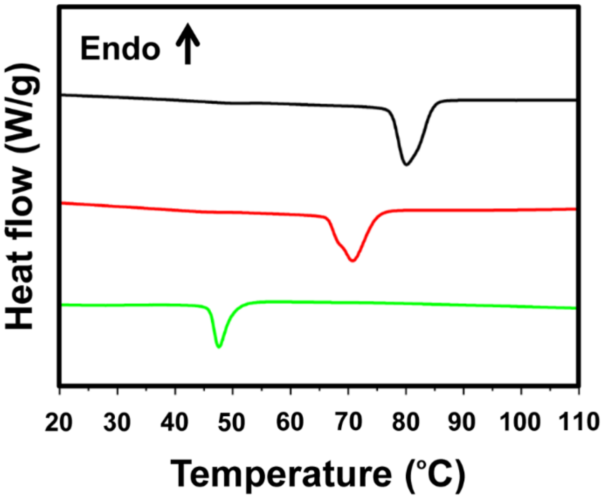

Figure S2. DSC curves of the cooling process of EVA9 (black), EVA14 (red), and EVA28 (green).

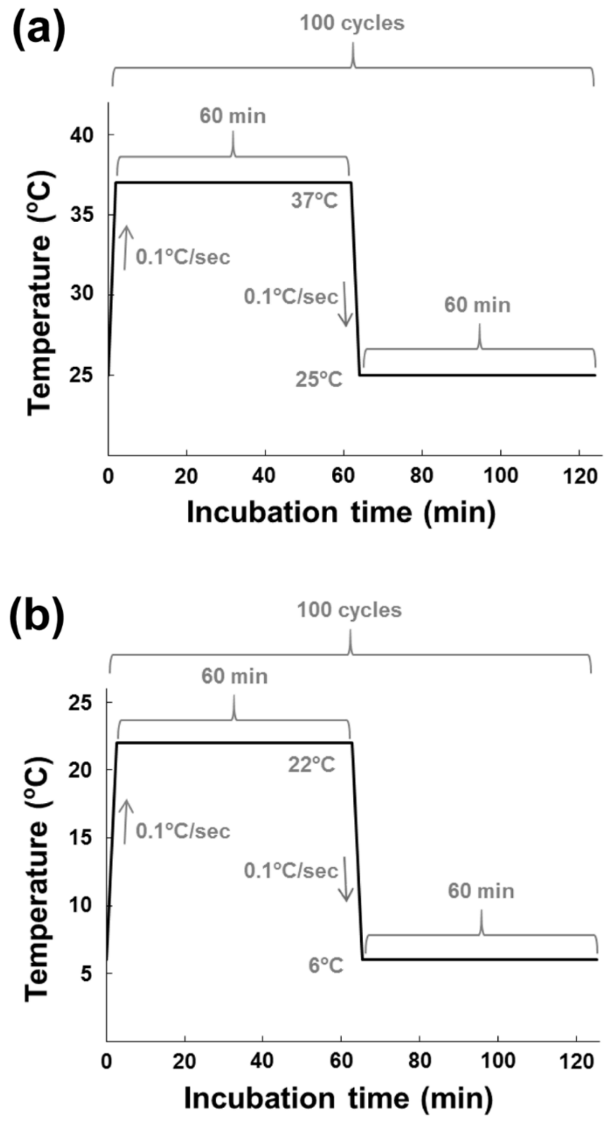

Figure S3. Thermal cycler protocols with repeated temperature fluctuations (a) between 25°C for 60 min and 37°C for 60 min, and (b) between 6°C for 60 min and 22°C for 60 min.

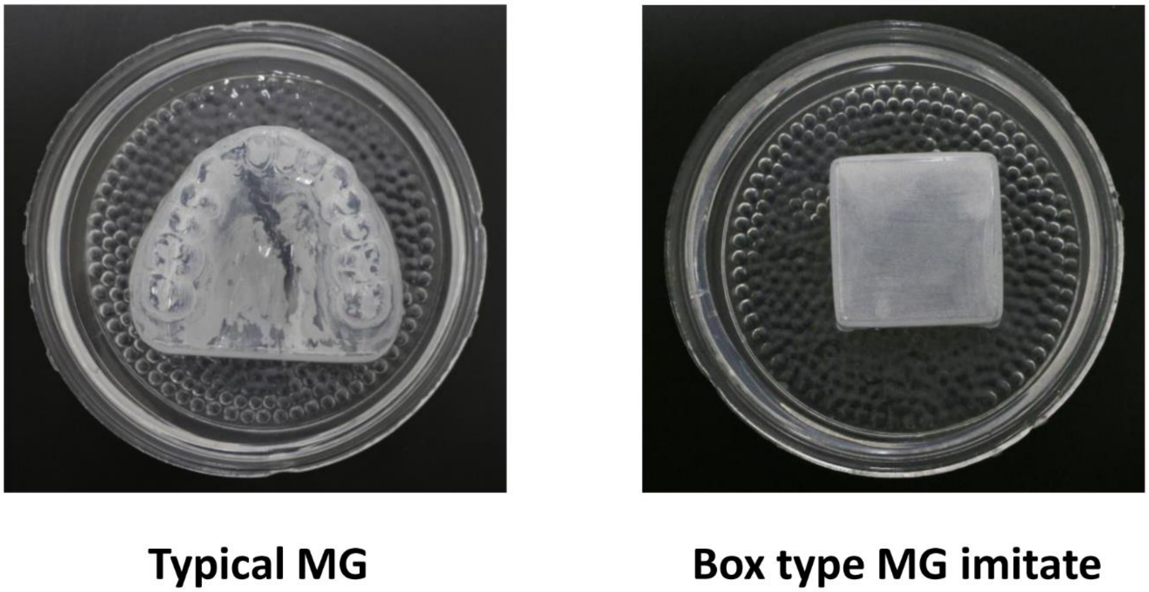

Figure S4. A typical MG (left) and a box-type MG imitate (right) prepared from clear-transparent Drufosoft® Type SQ EVA using the dental pressure laminate machine, Drufomat SQ.

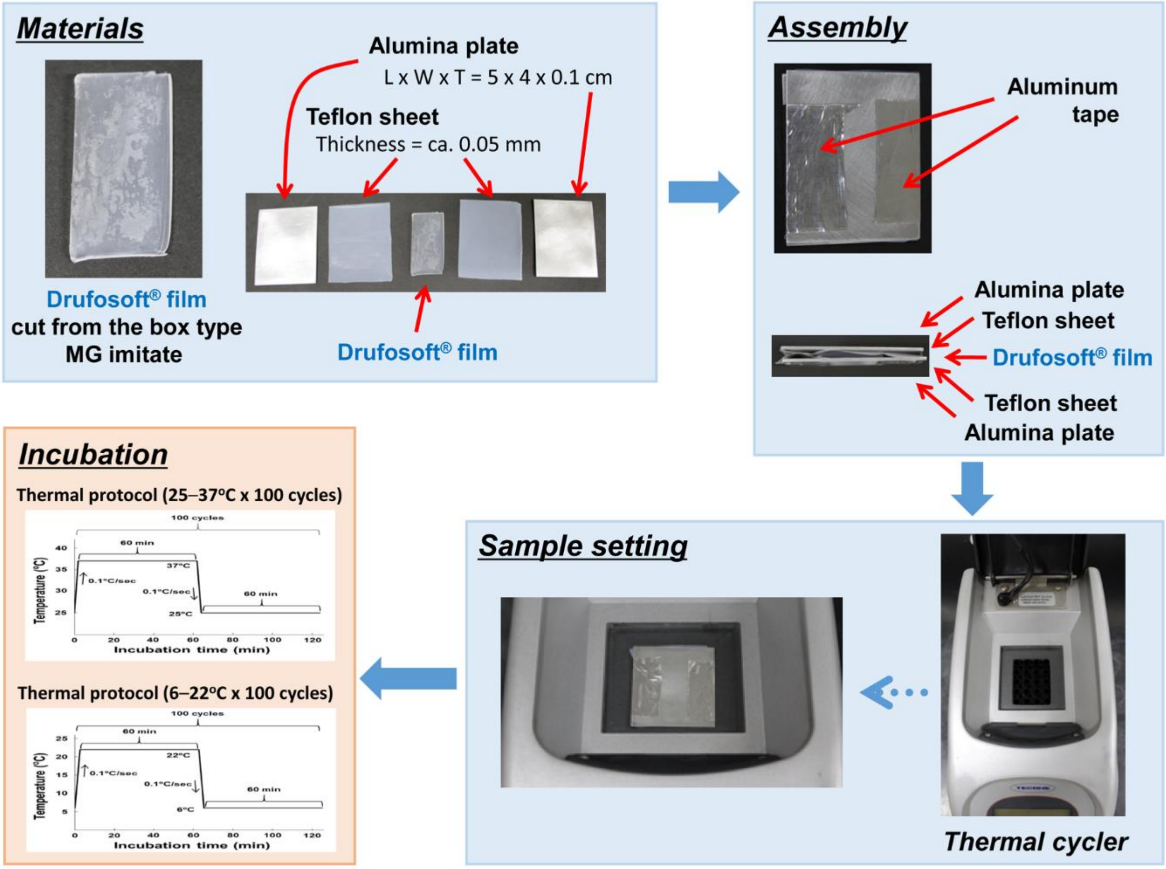

Figure S5. Schematic illustration of the preparation of thermal-treated samples for the repeated thermal cycle experiments.
